# Seq’ing the origins of cells in the developing spinal cord

**DOI:** 10.1016/j.jbc.2022.102602

**Published:** 2022-10-17

**Authors:** Prithviraj Rajebhosale, David A. Talmage

**Affiliations:** Section on Genetics of Neuronal Signaling, NINDS, National Institutes of Health, Bethesda, Maryland, USA

**Keywords:** Olig2, spinal cord, motor neuronN, oligodendrocyte, cell fate, trajectory mapping, zebrafish, pMN, hpf, hours postfertilization, MN, motor neuron, OL, oligodendrocyte, OPC, OL precursor cell, pMN, motor neuron progenitor, Shh, sonic hedgehog, TF, transcription factor

## Abstract

In the developing central nervous system, neurogenesis precedes gliogenesis; however, when and how progenitors are specified for a neuronal *versus* glial fate and the temporal regulation of this process is unclear. Progenitors within the motor neuron progenitor domain in the developing spinal cord give rise to cholinergic motor neurons and cells of the oligodendroglial lineage sequentially. In a recent study, Xing *et al.* used single cell RNA-seq to identify previously unknown heterogeneity of these progenitors in zebrafish and to delineate the trajectories that distinct pools of these progenitors take. These data help integrate existing evidence and inform new hypotheses regarding how populations of neural progenitors in the same spatial domain commit to distinct fates.

The vertebrate nervous system consists of diverse neuronal and nonneuronal cell types. Understanding how these cells are generated in a highly synchronized spatial and temporal manner has been a fascinating challenge for developmental biologists. Lineage tracing within the developing telencephalon (the anterior forebrain containing the cerebral lobes) has demonstrated that forebrain progenitors are clonally restricted to neuronal or glial fate prior to the onset of neurogenesis (1). However, the temporal regulation of this process remains a mystery. Studying these processes in the mammalian brain is technically challenging owing to the optical inaccessibility, complex evagination and folding of the cranial neural tube, and long cellular migrations following mitotic exit. The developing spinal cord, therefore, has been an excellent model system for defining the interaction of lineage-specific transcriptional regulators and extracellular morphogens in regulating progenitor diversity and fate.

In the developing spinal cord, distinct progenitor domains emerge along the dorsal-ventral extent of the central ventricular zone in response to varying concentrations of the morphogens sonic hedgehog (Shh) from the floor plate and bone morphogenic protein from the roof plate (2). The factors specifying these domains are evolutionarily highly conserved, making the developing zebrafish an excellent system to study the cell fate dynamics underlying progenitor specification and differentiation. Zebrafish embryos are optically transparent, allowing for live imaging of cells *in situ*, and can be genetically manipulated with relative ease. A particularly intriguing example of neurogenesis preceding gliogenesis in zebrafish occurs in one of the ventral-most progenitor domains, motor neuron progenitor (pMN), which sequentially gives rise to cholinergic motor neurons (MNs) and oligodendrocytes (OLs) (3). Both MNs and OL precursor cells (OPCs) are produced from a progenitor population that express the transcription factor *olig2*. Several competing models have been proposed to explain how, what appears to be a single population of progenitors gives rise to these distinct differentiated cell types. These include the common progenitor model where MNs and OPCs are born from the same progenitor cells, the heterogenous progenitor model where MNs and OPCs are generated from distinct progenitors (both of which express *olig2*) within pMN, and the progenitor recruitment model where, following MN production within pMN, a population of *olig2* nonexpressing progenitors migrate into pMN, initiate *olig2* expression, and then generate OPCs (4). While several studies have investigated the transcriptional regulators that are involved in MN *versus* OPC fate specification and the role of Shh in the process, how and when these distinct progenitors become specified and how their fate restriction is controlled remained elusive.

In their study published in JBC, Xing *et al.* (5) extended the single cell profiling of *olig2*+ cells from 28 h postfertilization (hpf) to 60 hpf to examine progenitor heterogeneity and fate. The authors found that pMN progenitors were heterogenous but generally clustered into cycling and noncycling progenitor clusters. Using pseudotime and cell trajectory analyses, they showed that these noncycling progenitors gave rise to neuroblasts fated to become MNs, as well as a population of progenitors that lay along a developmental trajectory from pMN progenitor to OPCs to OLs. Using transcription factor (TF) enrichment analysis, they identified several TFs that were enriched or depleted either in OPCs and pre-MNs. Among the TFs enriched in pre-MNs and strongly depleted in OPCs, *zfhx3* is involved in cholinergic MN identity in *Caenorrhabditis elegans*, whereas sox11b was previously shown to be involved in MN genesis after spinal cord injury, indicating that their enrichment in pre-MNs might be involved in shaping MN identity (6, 7). The identification of *myt1a* as a pre-MN–enriched TF was curious, as myt1 has been shown to be critical in regulating proliferation and differentiation of OPCs (8). Xing *et al.* generated *myt1a/b* KO zebrafish and found that these fish indeed had severe MN deficits, indicative of damaged and/or undifferentiated MNs. However, the authors did not find any noticeable differences in numbers of OPCs, indicating that myt1 might not be critical for OL lineage cells and revealing that MN terminal differentiation is not a prerequisite for OPC/OL production. Whether myt1 is a factor necessary to specify MN identity or maintain it, or plays some other role to direct neuronal morphogenesis, remains to be seen.

The current model of OPC production involves migration of dorsally located progenitors into the pMN domain, which initiate olig2 expression and, following a developmentally timed Shh surge, initiate *nkx2-2* expression and differentiate into OPCs fated to become OLs (Figure 1; 9, 10). Xing *et al.* identified an *nkx2-2*-negative pre-OPC population that might represent this migratory population captured at a point after the initiation of *olig2* expression but prior to *nkx2-2* expression. Interestingly, this population is present in the pMN at 28 hpf, during which time MN production is still ongoing. Previous studies have shown that a collaborative increase in Notch and Shh signaling in pMN progenitors leads to OL-fated OPC production (10); however, what sets these progenitors along this path is unclear. One possibility is that the pre-OPC migratory progenitors undergo genomic restructuring upon entering the pMN and initiating olig2 expression. There is evidence that in OPCs, olig2 directs chromatin remodeling complexes to genes poised for expression as these OPCs transition to their final OL fate. A better understanding of the identity and genomic structure of these progenitors prior to migration into the pMN might elucidate how these progenitors become restricted to an OPC fate upon entering the pMN. While a surge in Notch-Shh signaling is required for OL-fated OPC production, slightly lower levels of Notch signaling lead to the specification of NG2^+^ OPCs that remain as precursors. In contrast, NG2 glia in the central nervous system retain a strong proliferative potential even into adulthood (11). The authors also identified a population of cycling pMN progenitors in which expression of the lamin B receptor (*lbr*) was upregulated. Deleting lbr in rat neural stem cell cultures resulted in spontaneous differentiation of the stem cells into astrocytes and a few NG2^+^ glia even in the presence of stem cell medium. It would be interesting to know if the presence of high Shh with or without olig2 expression could direct the fate of *lbr*-KO neural stem cells to an OPC fate.

**Figure 1:**
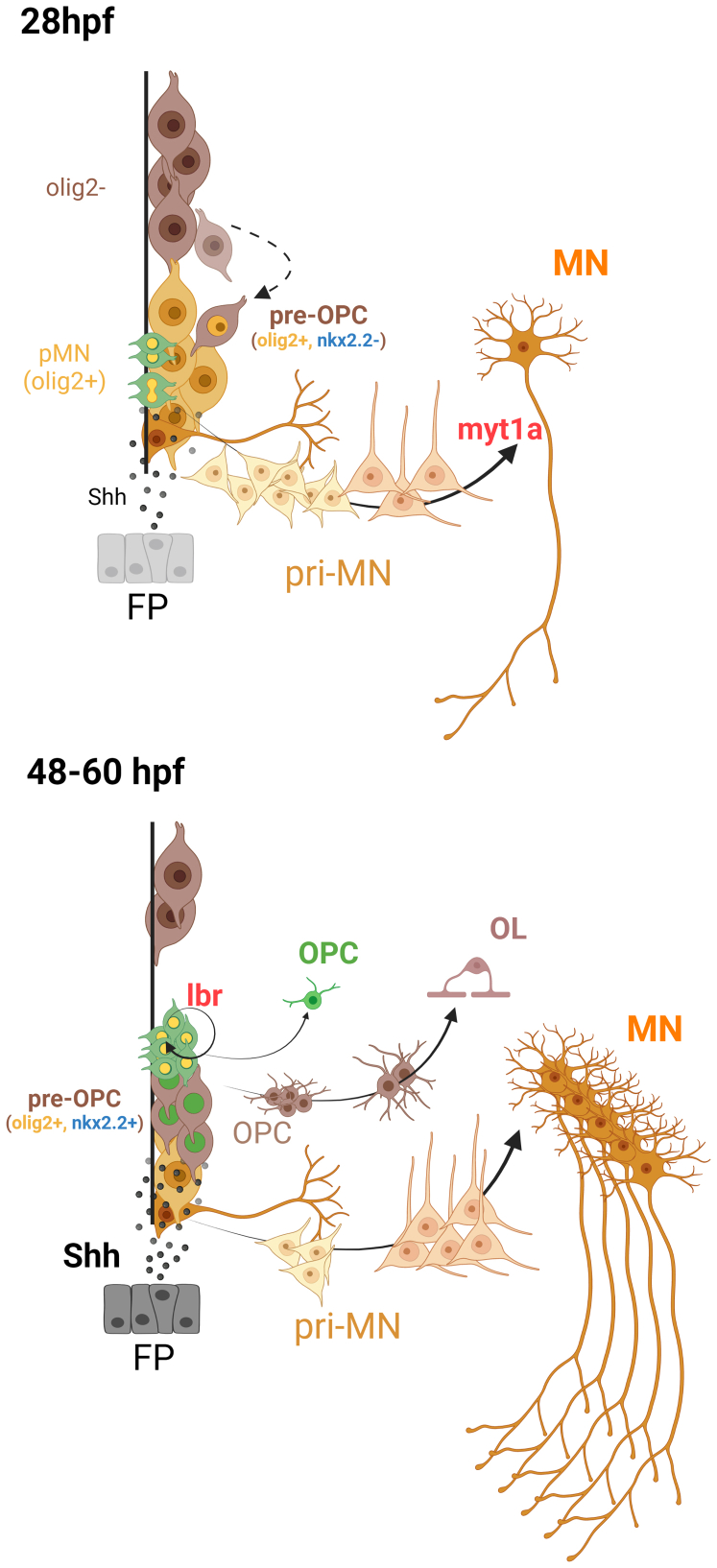
**Proposed model for mechanisms involved in sequential generation of motor neurons and oligodendrocytes in pMN.***Top*, at 28 hpf, olig2^+^ progenitors and radial glia (*yellow* cells) in the pMN generate motor neuron–fated neuroblasts (pri-MNs) which differentiate into MNs. Xing *et al.* identify myt1a as a novel regulator of this terminal differentiation. Cycling olig2^+^ progenitors (*green* cells with *yellow* nuclei) give rise to more MN-fate–restricted progenitors. As MN progenitors are depleted, olig2^−^ progenitors (*brown* cells) migrate into the pMN and initiate olig2 expression. At the levels of Shh (*black dots*, secreted from the floor plate; FP) present in the pMN at this stage, these cells would not be expected to express nkx2.2. Xing *et al.* identify a population of olig2^+^nkx2.2^−^ oligodendrocyte-fated progenitors. *Bottom*, these OL-fated pre-OPCs then differentiate into mature OLs and express nkx2.2 as Shh levels rise, around 36 hpf. Meanwhile, there remains a population of cycling olig2^+^ progenitors in the pMN that we propose might be the source of an OPC population that is not fated to differentiate and are maintained as NG2^+^ glia. Artwork created with BioRender. hpf, hours postfertilization; MN, motor neuron; OL, oligodendrocyte; OPC, OL precursor cell; pMN, motor neuron progenitor; Shh, sonic hedgehog.

The transcriptome can reveal a current state of a cell or what a cell might be getting ready to do. However, simultaneous profiling of chromatin accessibility informs the functional bounds of the cell and potentially also informs future states of which a cell might be capable. Multi-omic technologies have been developed that allow simultaneous profiling the transcriptome and regions of open chromatin at the single cell level. Applying such a strategy to the current paradigm might provide further answers to questions regarding how heterogeneity within pMN progenitors interacts with the extracellular environment to instruct distinct fates.

## Conflict of interest

The authors declare that they have no conflicts of interest with the contents of this article.
